# *Giardia duodenalis* (Styles, 1902) in Cattle: Isolation of Calves with Diarrhoea and Manure Treatment in the Lagoon Presented as Risk Factors in Latvian Herds

**DOI:** 10.3390/microorganisms11092338

**Published:** 2023-09-18

**Authors:** Maira Mateusa, Maija Selezņova, Margarita Terentjeva, Gunita Deksne

**Affiliations:** 1 Institute of Food Safety, Animal Health and Environment BIOR, 1076 Riga, Latvia; maija.seleznova@bior.lv (M.S.); margarita.terentjeva@lbtu.lv (M.T.); 2 Faculty of Veterinary Medicine, Latvia University of Life Sciences and Technologies, 3001 Jelgava, Latvia; 3 Faculty of Biology, University of Latvia, 1004 Riga, Latvia

**Keywords:** epidemiology, protozoan, zoonosis, prevalence, assemblages, risk factors, calves, diarrhoea

## Abstract

*Giardia duodenalis* is a waterborne zoonotic protozoan that causes gastrointestinal tract inflammation in humans, cattle, and other animals. The aim of the present study was to estimate the prevalence and potential risk factors for *Giardia* infection in cattle in Latvia. During 2020–2021, a total of 973 individual faecal samples from cattle aged from 1 day to 12 years old, from 32 cattle herds, were tested for *Giardia* cyst presence with immunofluorescence staining followed by *Giardia* assemblage differentiation targeting *beta-giardin* gene. Using a questionnaire, information was collected to estimate the potential risk factors for *G. duodenalis* infection in cattle herds. *Giardia* was found in 8.4% of the examined cattle with a mean intensity of 5756 cysts per gram of faeces. The highest prevalence was observed in the 0 to 3-month-old calves (16.4%). At least one *Giardia* shedding animal was found in 27 herds with an overall prevalence of 84.4%. Significantly higher prevalence was found for cattle infected with *G. duodenalis* assemblage E compared to that infected with assemblage A: 88.7% and 11.3%, respectively. Protective factors such as age and rodent control and change of shoes were found to be significant for *Giardia* infection, while isolating calves for diarrhoea and water bodies (ponds/lakes) in pasture were potential risk factors in Latvian cattle.

## 1. Introduction

*Giardia duodenalis* (Styles, 1902) (syn. *G. lamblia* or *G. intestinalis*) is a waterborne and foodborne zoonotic protozoan, which infects the gastrointestinal tract of humans and animals, causing self-limiting diarrhoea [1,2]. Currently, eight *G. duodenalis* assemblages are molecularly differentiated (A to H) from which assemblage E is specific to cattle [1]. The zoonotic assemblage A can also infect cattle, while assemblages B and C have been detected in cattle, but there is not enough evidence to prove that these assemblages may cause true infections [1,2,3]. The faecal–oral transmission of cysts occurs either through direct contact with infected hosts or indirectly from contaminated food, feed, water, or equipment [1,4].

In cattle, *Giardia* causes inflammation in the small intestine, which leads to diarrhoea, maldigestion and malabsorption, resulting in poor growth and weight loss [1,5]. Clinical giardiasis is observed in calves under six months of age, with calves under one month old being affected more severely [6]. Adult cattle, especially around the periparturient period, can shed cysts without showing any clinical symptoms [6,7,8]. A causal association between shedding cysts and diarrheal disease is not always convincing, because diarrhoea in ruminants could be caused by a combination of other infections and inadequate husbandry practices [4,9]. It is worth noting that the diverse clinical courses of the disease may be related to the presence of virulence factors among different *Giardia* strains, the nutritional status of the host, gut microbiota, coinfections with other pathogens, and host immune responses [1,4,10].

The infective dose can be as low as 10 cysts, and young calves can shed as much as 10^6^ cysts per gram of faeces; this, in combination with the extended cyst survival in the organic matter and water, places calves as an important source of environmental contamination and could be a potential public health hazard [6,11,12,13]. It has been shown that faeces from livestock could be a threat to recreational water sources [11]. Drinking water supplies may become contaminated either by direct faecal deposition when pastures are in the vicinity of surface waters or indirectly by runoff from fields, where improperly treated manure is applied or effluent from herd spills over [11,14].

In Europe, the prevalence of *Giardia* in cattle is reported to be 35.1%, with the highest prevalence found in neonatal calves (60.7%) and the lowest in heifers (7.8%), with assemblages A, E and B reported [6]. Several risk factors have been associated with *Giardia* infection in cattle, such as age, irregular cleaning of maternity pens, prolonged contact with a dam, and calf grouping [11,15,16,17]. Regular disinfection, high-pressure cleaning, and empty periods between the introduction of new calves could potentially decrease the infection risks [15,16,17].

In Latvia, giardiasis is an underdiagnosed and underreported disease in humans, and the prevalence was reported as high as 7.2% in humans under the age of 17 years [18]. Giardiasis is not included in the list of notifiable animal diseases [19], and the lack of studies on *Giardia* in the Latvian animal population indicates the gap of knowledge about the *Giardia* and the epidemiology in Latvian cattle herds.

The aim of the present study was to estimate the prevalence of *G. duodenalis* and to estimate the potential risk factors associated with *Giardia* infection in Latvian cattle herds.

## 2. Materials and Methods

### 2.1. Study Design

Sample size was calculated based on a Latvian cattle population size of 395,320 (Agricultural Data Centre Republic of Latvia, accessed on 1 January 2020). To minimise sampling bias, sampling was stratified to the counties of Latvia, and potential herd owners were contacted for possible sampling. The main inclusion criteria were herds with different management systems (such as untethered and tethered management types) and size (from large industrial herds to small family-owned herds). Up to 45 faecal samples per herd were collected—15 samples from three different age groups (0–3 months, 4–24 months, and older than 24 months old). In cases where there were not enough animals in a specific age group, samples from all animals were collected.

Samples were collected from ground right after defecation. Information about faecal consistency was also noted (diarrhoea or no diarrhoea). Samples were put in single-use plastic containers, labelled, transported to the laboratory, and stored at +4 °C until further testing.

### 2.2. Questionnaire

A questionnaire was designed to gather information about herds and herd management. It was divided in five different categories: calf management (calving place, presence of diarrhoea, calf grouping), walking area and pastures (start and end of pasture season, drinking water in pastures), herd management (cleaning of herd and sleeping area, disinfection, and pests), feed, and the surrounding environment around the herd. A questionnaire in Latvian was filled by interviewing farmers during sample collection (Supplementary File S1). Written consent was acquired from owners to collect faecal samples from the ground and data gathering.

### 2.3. Immunofluorescent Microscopy Analysis

The sample was prepared by saturating one gram of material in a sodium chloride (NaCl) flotation solution. One gram of faeces was subjected for one flotation and multiple centrifugation steps, which yielded 2 mL of concentrated material for subsequent analysis [20]. A purified and mixed sample (10 μL) was stained with FITC-labelled *anti-Cryptosporidium/Giardia* monoclonal antibodies (AquaGlo^TM^, Waterborne Inc., New Orleans, LA, USA) according to the manufacturer’s instructions. Enumeration was performed by counting all bright-green cysts with typical morphology and size, with each detected cyst representing 200 cysts per gram (CPG) as described by Maddox-Hyttel et al. [15].

### 2.4. Identification of Giardia Cysts at the Assemblage Level by PCR/RFLP

Genomic DNA was extracted from the pellets obtained after centrifugation of the 2 mL purified faecal sample using the DNeasy PowerSoil Kit (QIAGEN, Hilden, Germany) according to the manufacturer’s instructions. Elution was completed with 80 μL of Solution C6 (i.e., the elution buffer of the DNeasy PowerSoil Kit). Identification of *Giardia* cysts at the assemblage level by PCR/RFLP was used, and 10 μL of each DNA sample was subjected to nested-PCR amplifications targeting the *beta-giardin* gene and the subsequent digestion of the PCR fragments with the restriction endonuclease HaeIII as previously described by the European Reference laboratory of Parasites [21]. Nuclease-free water and *Giardia* genomic DNA were used as negative and positive controls. The PCR products and PCR-digested fragments were run on capillary electrophoresis (QIAxel Advances, QIAGEN, Hilden, Germany). The sizes of the fragments obtained with the two consecutive PCR amplifications were 723 and 511 base pairs (bp), respectively. Using the PCR/RFLP technique, it is possible to distinguish *G. duodenalis* assemblage A, B, C, D, E, F and G based on the number and size of the fragments obtained by digestion of the 511 bp fragment of the *beta-giardin* gene with the HaeIII enzyme [22,23].

### 2.5. Statistics

Medians and means were calculated to obtain data for the counted cysts per gram from age groups and herd sizes. The 95% confidence intervals were calculated for point estimated proportions (proportion of *Giardia* positive animals) according to Wilson (1927) [24], using the Mid-p Exact on OpenEpi (OpenEpi: Open Source Epidemiologic Statistics for Public Health, Version. 3.01., www.OpenEpi.com, accessed on 1 May 2023) [25]. Two-tailed *p* < 0.05 was considered statistically significant. A herd was considered positive if at least one animal excreted a *Giardia* cyst. An animal was considered positive if the faecal sample contained at least one *Giardia* cyst.

In risk factor analysis, a *Giardia* microscopic result (“GiardiaYN”) was considered as a dependent variable. For the identification of potential risk factors—individual animal and herd-level factors—generalised linear mixed modelling fit by maximum likelihood (Laplace approximation) was performed using R and RStudio version 4.2.2 [26] by applying the package lme4 [27], i.e., using the “glmer” function and assuming a binomial distribution, including individual herd identification number (“HerdID”) as a random effects variable. Apart from animal age, sex, breed, and the presence of diarrhoea (present or not), all factors were assessed at the herd level.

Age was expected to be an important effect-modifying explanatory variable; therefore, data on age (in days) were included into each of the models and calculated to identify putative risk or protective factors for *Giardia* presence.

Variables, which were significant or tended to be significant (0.05 ≤ *p* < 0.1) in the initial model, were included in the final generalised linear mixed model for potential risk or protective factor determination for *Giardia* infection in cattle. By optimising the model with a stepwise elimination of variables, which did not cause an increase in Akaike information criterion (AIC), a final linear mixed model was generated.

## 3. Results

From March 2020 to March 2021, a total of 45 herd owners were contacted, out of which 32 responded and were visited during this study. A total of 973 individual faecal samples (10–45 samples per herd) were collected from cattle aged from 1 day to 12 years old. Average age of animals sampled was 720.9 days (1–4433 days), with the highest number of samples collected from animals older than 24 months (Table 1).

### 3.1. Overall Prevalence of Giardia Duodenalis

The overall prevalence of *Giardia* in cattle was 8.4% (N = 82; 95%CI: 6.8–10.3), with the mean CPG 5756 (median 600; min 200; max 62,600). The highest proportion was observed in 0 to 3-month-old calves (Table 1), and a significant difference in the prevalence of *G. duodenalis* was observed between the first age group (0–3-months) and the other age groups.

At least one *Giardia* cyst-shedding animal was found in 27 visited herds, with the overall prevalence being 84.4% (95%CI: 67.8–93.6). The lowest prevalence of 42.9% (95%CI: 15.7–75.0) was observed in herds with 151–250 animals, which was followed by 90% in herds less than 150 animals (95%CI: 54.1–100) and 100% prevalence in herds with 251–500 and more than 501 animals (95%CI: 59.6–100; 62.8–100). The highest mean CPG (18,250) was observed in herds with 151–250 animals and in the age group of 0–3-month-old animals (Table 2). Even though a higher proportion of diarrhoea in *Giardia*-shedding animals was found in 251–500 animal-sized herds, in both 0–3- and 4–24-month-old animal age groups, no statistical significance was observed (*p* > 0.05).

A higher prevalence of 11.7% was observed in males (95%CI: 6.9–18.7) than in females (8.0%, 95%CI: 6.3–10.0) cattle, but no statistical significance was observed (*p* = 0.2389). A higher mean CPG was observed in male cattle—8071.4 (median 2200; min 200; max 62,600) than in female cattle—5279.4 (median 600; min 200; max 56,600), but no statistical significance was observed (*p* > 0.05)

Samples were collected from 12 cattle breeds, but no statistical difference was observed between cattle breeds (*p* > 0.05).

### 3.2. Giardia Assemblage Identification

*Giardia* DNA was successfully amplified from 62 (75.6%) of the 82 faecal samples from 27 herds which were microscopically positive. Overall, two *Giardia* assemblages were detected—A (11.3%, *n* = 7) and E (88.7%, *n* = 55). Assemblage E was prevalent in all age groups, while assemblage A was prevalent in age groups 4–24 months (*n* = 5 animals) and >24 months (*n* = 2 animals). Meanwhile, assemblage E was prevalent in all herd size groups, while assemblage A was prevalent in herds with 251–500 (*n* = 2 herds) and more than 500 animals per herd (*n* = 5 herds).

### 3.3. Models

Age had a statistically significant (*p* < 0.05) effect on *Giardia* in animals, which was calculated by generalised linear mixed modelling fit by maximum likelihood (Laplace approximation) (Table 3).

Therefore, it was concluded that *Giardia* prevalence decreased in older animals. In the first generalised linear mixed model, age in days (referred as “Age”) was used as an effect-modifying variable and the individual herd identification number (referred as “HerdID”), as a random effect variable revealed that statistically significant (*p* < 0.05) risk factors included the following: animals that can leave herd territory (either have pastures or separate walking area), isolating calves with diarrhoea, having a walking area, drinking water in pasture, water bodies (ponds, lakes, rivers) in pasture, and manure treatment in lagoon. However, pasture start in May, manure kept in an open space, rodent control with poison, and change of shoes for visitors and cats as pet animals were statistically significant (*p* < 0.05) protective factors for *Giardia* prevalence in animals (Table 3).

Further analyses were conducted for all risk or protective factors that were significant at a level of *p* < 0.1, in addition to “Age” (Table 3) in bivariable generalised linear mixed models, including “Age” as an effect modifier and “HerdID” as a random effect variable for the presence of *Giardia* in animals (Table 4). The isolation of calves with diarrhoea was excluded from further analysis, because that could potentially be a consequence of exposure to *Giardia* rather than a risk.

## 4. Discussion

Overall, the prevalence of *Giardia* in cattle was 8.4%. The highest prevalence of 16.4% and the mean CPG of 8109.4 was observed in animals under 3 months of age. The age was a protective factor, and older cattle were less likely infected with giardia (*p* < 0.05). Results obtained from Scotland, Denmark, and Spain show similar tendencies, where weaning and suckling calves had a higher overall *Giardia* prevalence compared to other age groups [15,28,29]. Older cattle from 4–24 and above 24-month-old age groups shed *Giardia* cysts with the mean CPG 1284.2 and 1780.0, respectively; therefore, older cattle could be a potential source of infection for not only younger cattle but also for humans and the environment [30].

No statistical significance was observed between both sexes in our study, which shows that sex most likely is not a predisposition for *Giardia* infection in cattle. However, male cattle had higher *Giardia* prevalence than female cattle, which could be due to different management practices. Namely, male cattle are usually kept until they are grown enough to be sold off or slaughtered for meat production, whereas female cattle are typically kept for further breeding [31]. It is also worth noting that there were fewer samples collected from male cattle during this study; therefore, these conclusions may not be definite. There has been a study showing better immune response to pathogens in female calves in the pre-pubertal stage [32], which could affect immune reaction for *Giardia* infection; however, this should be further investigated.

Prevalence between sampled herds varied 42.9–100.0%, but the highest overall prevalence was observed in two herd size groups: 251–500 and above 500 animals per herd (100.0%). In larger size herds, due to overcrowding within the herd, close contact with other infected animals, or frequent change of animals within an animal age group, there could be an increase in possible pathogen transmission between animals [17].

Two *Giardia* assemblages but no mixed infections were observed in this study. Assemblage E was most predominant in all cattle age groups and all herd sizes, which was also observed in other studies conducted in Europe [6]. Assemblage E has the potential to decrease immune response and immune cell migration, therefore leading to a chronic course of this disease in cattle [33]. Zoonotic assemblage A was observed in cattle older than 4 months; however, there are other studies showing that this assemblage is often found in younger cattle [16,34,35]. Therefore, calves are assumed to be the main potential zoonotic source of human infection. Our study shows that older cattle also have the potential to spread cysts into the environment.

There was no statistical significance observed between *Giardia* and diarrhoea in cattle in this study (*p* > 0.05); however, a higher proportion of diarrhoea was observed in 0–3-month-old calves, especially from 251–500 animal-sized herds, with similar findings observed in other studies conducted in Denmark, Germany, France, Italy, and the United Kingdom [15,16]. Even if *Giardia* does not potentially cause acute diarrhoea in calves, it can cause chronic, intermittent diarrhoea, which could limit cyst detection. Chronic giardiasis reduces intestinal surface area, consequently reducing intestinal enzyme activity, resulting in weight loss and lower immunity, which could lead to other enteric pathogen activity (such as *Cryptosporidium* spp., rotavirus, Coronavirus, *Eimeria* spp., etc.) [5,36,37].

In the initial model, several putative protective factors were observed, such as rodent control with poison, change of shoes for visitors, and cat presence in the herd (Table 3). Rodents can act as mechanical or direct vectors of *Giardia*, and it has been shown that they can potentially carry assemblages A and B [38,39]; therefore, rodent control acts as a protective factor and may reduce *Giardia* cyst prevalence in herds from the vectors. The lower risk due to changing visitor shoes shows that the simple introduction of epidemiological safety regulations within a herd could minimise the transmission routes of *Giardia* and other pathogens into the herd [40].

Change of shoes for visitors does not have a biological explanation; however, visitors on the herd could be a way for *Giardia* and other pathogens to be introduced into the herd if no proper epidemiological safety precautions are put in place [40].

In the final model, factors like animals not being able to leave herd premises, the pasture season starting in May, no access to pastures, and the manure being kept in either open pits or piles next to the herd were identified as putative protective factors. Starting a pasture season in May and having no pasture appeared to be putative protective factors, although they are contradictory. Animals that have a grazing season could have a potentially lower immune system due to lower iron, folic acid, and vitamin B_12_ levels, and they could also have a higher chance of exposure to endoparasites; however, cyst load in the pastures is minimised because of the large pasture area [41,42,43]. In herds where cattle are kept indoors only and do not have access to pastures, the animals are provided with the necessary nutrients and balanced diet, therefore increasing immunity in cattle. However, there is a higher likelihood of crowding, which may increase the direct transmission of pathogens from cattle to cattle and increase the environmental load with infective cysts [1,43]. Manure kept in open pits or piles as a protective factor does not have any biological explanation, because even though they do produce initial heat in the central core that is needed to reduce protozoan pathogenicity, these conditions are not met in manure pile sides, and potential run-offs from these types of pits possibly contain viable pathogens, which are re-introduced into the herd and the environment [44,45].

In the initial model, several risk factors were observed: isolation of calves with diarrhoea; having a walking area; availability of drinking water in pastures; access to free water (ponds, lakes, rivers) in pastures; and manure treatment in a lagoon. Isolating calves with diarrhoea as a potential risk effect does not have any biological explanation, as removal of the infected calf from the rest of the group minimises exposure to cysts, since one infected calf can produce up to 10^6^ cysts per gram of faeces, and predicted infective dose can be as low as 10 cysts [1,46]. The walking area could minimise animal density on-premises; however, walking areas are rarely cleaned, therefore increasing potential environmental load with infective cysts, and potential environmental factors, such as exposure to direct sunlight, could reduce the infectivity of those cysts, which are exposed to sunrays [47]. Drinking water or access to water bodies (ponds, lakes, rivers) in pastures showed potential risk for cattle, therefore only confirming that water is one of the main *Giardia* transmission routes not only for animals but, potentially, also for humans [1,4].

Manure treatment with a lagoon (a closed pit, where manure goes through anaerobic processes) as a risk factor does not have any biological explanation, because lagoons could reduce *Giardia* cysts up to 100% [48], which could be a potential way to reduce not only cyst viability but also reduce potential environmental contamination with cysts if manure is used for field and crop fertilisation [49]. Using lagoons for manure processing could potentially reduce foodborne and waterborne risks for humans, reduce exposure to mechanical vectors—such as cats and rodents, as well as reduce manure/slurry run-offs into the environment around herds [45,50]. In the present study, all analysed herds used manure for field fertilisation, but most of the herds kept manure in either an open pile or open pit, which was shown to be a risk factor in the initial and the final model.

## 5. Conclusions

*G. duodenalis* is prevalent in Latvian cattle herds with the highest proportion observed in animals below 3 months of age, with cattle-specific assemblage E and zoonotic assemblage A found on most of the herds. Protective factors, such as age, change of shoes for visitors, pasture start in May, or no available pastures could decrease the risk of *Giardia* in the Latvian cattle population. However, open water bodies (ponds/lakes/rivers) have increased risk for giardia in cattle and also may increase risk of transmission for humans.

## Figures and Tables

**Table 1:** *Giardia duodenalis* prevalence, the proportion of diarrhoea in cyst-shedding animals, and cysts per gram per different animal age groups and herd size.

Factor		Total No. Analysed/Infected Animals	Prevalence (95%CI)	Mean CPG	Median CPG	Min-Max CPG	Proportion of Diarrhoea (95%CI)
Age group	0–3 months	324/53	16.4 (12.7–20.8) ^a^	8109.4	1600	200–62,600	32.1 (21.0–45.5)
	4–24 months	281/19	6.8 (4.3–10.4) ^b^	1284.2	400	200–9600	15.8 (4.7–38.4)
	>24 months	368/10	2.3 (1.4–5.0) ^c^	1780.0	200	200–15,800	0.0 (0.0–0.0)
Herd size	<150 animals	259/26	10.0 (6.9–14.3)	2938.5	600	200–24,200	23.1 (10.7–42.4)
	151–250 animals	207/10	4.8 (2.5–8.8)	14640.0	11700	200–56,600	10.0 (0.0–42.6)
	251–500 animals	219/23	10.5 (7.0–15.3)	6191.3	1000	200–62,600	47.8 (29.2–67.0)
	>501 animals	288/23	7.9 (5.3–11.7)	4643.5	400	200–55,000	4.5 (1.2–27.9)

CI, confidence interval; CPG, cysts per gram; ^a^—the prevalence of *G. duodenalis* was significantly (*p* < 0.05) higher than for 4–24, >24 months age groups; ^b,c^—difference between the prevalence for groups 4–24 and >24 was not significant.

**Table 2:** *Giardia duodenalis* prevalence and proportion of diarrhoea of cyst-shedding animals per different age groups in different herd sizes.

Age Group	0–3 Months			4–24 Months			>24 Months		
Herd Size, Number of Animals	Total No Analysed/ Prevalence (95%CI)	Proportion of Positive Findings in Animals with Diarrhoea (95%CI)	Mean CPG	Total No Analysed/ Prevalence (95%CI)	Proportion of Positive Findings in Animals with Diarrhoea (95%CI)	Mean CPG	Total No Analysed/ Prevalence (95%CI)	Proportion of Positive Findings in Animals with Diarrhoea (95%CI)	Mean CPG
<150 animals	77/22.1 (14.2–32.6)	32.3 (17.2–58.8)	3988.2	76/5.3 (14.2–32.6)	0.0 (0.0–54.6)	1850.0	106/4.7 (1.7–10.8)	0.0 (0.0–48.9)	240.0
151–250 animals	54/14.8 (7.4–26.9)	12.5 (0.1–49.2)	18,250.0	41/2.4 (0.0–5.4)	0.0 (0.0–83.2)	200.0	112/0.9 (0.0–5.4)	0.0 (0.0–83.2)	200.0
251–500 animals	82/19.5 (12.3–29.5)	50.0 (28.0–72.0)	7625.0	58/8.6 (3.3–19.0)	60.0 (22.9–88.4)	880.0	79/2.5 (0.2–9.3)	0.0 (0.0–71.0)	8000.0
>501 animals	111/10.8 (6.1–18.1)	16.7 (3.5–46.0)	7833.3	106/8.5 (4.3–15.5)	0.0 (0.0–34.5)	1377.7	71/2.8 (0.2–10.3)	0.0 (0.0–71.0)	200.0

CI, confidence interval, CPG, cysts per gram.

**Table 3:** Fixed effects in generalised linear mixed models to determine potential risk factors for *Giardia* presence in Latvian cattle. Data were analysed by bivariable generalised linear mixed modelling including age in days (“Age”) as an effect modifier and herd identification number (“HerdID”) as a random effects variable in modelling. The Akaike information criterion (AIC) was used to characterise the relative model quality. Only models with statistically significant explanatory variables (*p* < 0.05) or variables tending to be significant (0.05 ≤ *p*< 0.1) in addition to “Age” are displayed.

Model (AIC, Model Fit)	Variable	Odds Ratio (95%CI)	Z Value	*p*-Value
1 (527.4)	(intercept)	0.1 (0.0–0.1)	−16.5	*p* < 0.01
	Age	0.4 (0.3–0.6)	−5.6	*p* < 0.01
2 (524.8)	(Intercept)	0.04 (0.0–0.1)	−11.2	*p* < 0.01
	Age	0.1 (0.07–0.4)	−3.9	*p* < 0.01
	Can animal leave herd: no (ref)			
	Can animal leave herd: yes	1.8 (1.0–3.2)	2.1	0.04 **
3. (524.9)	(Intercept)	0.06 (0.04–0.1)	−15.7	*p* < 0.01
	Age	0.4 (0.3–0.6)	−5.5	*p* < 0.01
	Calf isolation with diarrhoea: no (ref)			
	Calf isolation with diarrhoea: yes	1.7 (1.0–2.7)	2.2	0.03 **
4. (524.4)	(Intercept)	0.05 (0.04–0.1)	−14.6	*p* < 0.01
	Age	0.4 (0.3–0.6)	−5.7	*p* < 0.01
	Walking area: no (ref)			
	Walking area: yes	1.7 (1.1–2.7)	2.3	0.02 **
5. (527.1)	(Intercept)	0.2 (0.1–0.4)	−3.8	0.000104 ***
	Age	0.4 (0.3–0.7)	−5.7	*p* < 0.01
	Pasture season start: April (ref)			
	Pasture season start: May	0.4 (0.1–1.1)	−1.7	0.08 *
	No pasture	0.3 (0.1–0.9)	−2.2	0.03 **
6. (524.8)	(Intercept)	0.06 (0.03–0.1)	−11.4	*p* < 0.01
	Age	0.4 (0.3–0.6)	−5.6	*p* < 0.01
	Drinking water in pasture: no (ref)			
	Drinking water in pasture: yes	1.9 (1.1–3.6)	2.2	0.03 **
	No pasture	1.0 (0.6–1.9)	0.1	0.90
7. (526.8)	(Intercept)	0.06 (0.05–0.09)	−16.7	*p* < 0.01
	Age Animals can access free water in pasture: no (ref)	0.4 (0.3–0.6)	−5.6	*p* < 0.01
	Animals can access free water in pasture (yes)	1.6 (0.9–2.8)	1.7	0.09 *
8. (527.3)	(Intercept)	0.1 (0.07–0.2)	−9.6	*p* < 0.01
	Age	0.4 (0.3–0.6)	−5.5	*p* < 0.01
	Manure in closed space (ref)			
	Manure in open space	0.6 (0.3–1.0)	−2.0	0.04 **
	Manure kept in pile	0.6 (0.3–1.2)	−1.4	0.16
9. (525.9)	(Intercept)	0.04 (0.01–0.1)	−6.2	*p* < 0.01
	Age	0.4 (0.3–0.6)	−5.6	*p* < 0.01
	Manure treatment: fermentation (ref)			
	Manure treatment: lagoon	2.7 (0.9–8.1)	1.8	0.07 *
	Manure treatment: no treatment	1.6 (0.6–4.7)	0.8	0.37
10. (526.0)	(Intercept)	0.1 (0.07–0.14)	−12.2	*p* < 0.01
	Age	0.4 (0.3–0.6)	−5.6	*p* < 0.01
	Rodent control—cat (ref)			
	Rodent control—no control	0.7 (0.2–3.2)	−0.4	0.66
	Rodent control—poison	0.6 (0.3–0.9)	−2.4	0.02 **
11. (525.9)	(Intercept)	0.1 (0.06–0.14)	−11.8	*p* < 0.01
	Age	0.4 (0.3–0.6)	−5.6	*p* < 0.01
	Change of shoes for visitors	0.6 (0.4–1.0)	−1.9	0.05 *
12. (525.3)	(Intercept)	0.1 (0.07–0.16)	−10.3	*p* < 0.01
	Age	0.4 (0.3–0.6)	−5.7	*p* < 0.01
	Pet animals: Cat	0.6 (0.4–1.0)	−2.1	0.03 **

CI, confidence interval; AIC, Akaike information criterion; ref., reference; * *p* < 0.1; ** *p* < 0.05; *** *p* < 0.001.

**Table 4:** Fixed effects in generalised linear mixed models to determinate potential risk and protective factors for *Giardia duodenalis* prevalence in cattle. The Akaike information criterion (AIC) was used to characterise the relative model quality.

Model (AIC Model Fit)	Predictors	Odds Ratios	95% CI	Z-Value	*p*-Value
Final (521.2)	(Intercept)	0.2	0.1–1.0	−2.0	0.04
	Age	0.4	0.3–0.6	−5.7	<0.001
	Can animals leave herd: Yes	2.2	1.1–4.7	2.2	0.03
	Pasture season start: May	0.2	0.1–0.8	−2.4	0.02
	No pastures	0.3	0.1–0.9	−2.2	0.03
	Manure kept in open pit	0.5	0.3–0.9	−2.3	0.02
	Manure kept in pile	0.3	0.1–0.7	−2.7	0.01

AIC, The Akaike information criterion; CI, confidence interval.

## Data Availability

The data presented in this study are available on request from the corresponding author.

## Supplementary Materials

The following supporting information can be downloaded at: https://www.mdpi.com/article/10.3390/microorganisms11092338/s1, File S1: Questionnaire.
