# Supplementary material for: Giardia duodenalis (Styles, 1902) in Cattle: Isolation of Calves with Diarrhoea and Manure Treatment in the Lagoon Presented as Risk Factors in Latvian Herds
Source: Microorganisms. 2023 Sep 18;11(9):2338. doi: 10.3390/microorganisms11092338 (PMC10537315; doi:10.3390/microorganisms11092338)
Supplement: Supplementary file 1 [file microorganisms-11-02338-s001.zip › microorganisms-2598906-supplementary.pdf]

ID NR__________ Date________

Consent form

Farm owner is kindly asked to fill out questionnaire. Questionnaire is anonymous, no sensitive data about farm will be shared with the public.

I agree, that freshly defecated feaces are collected from cows on my farm. ________________________/date, signature/

☐ E-mail for results____

Farm number:____

Herd number:____

ID NR___________ Date_________

Questionnaire about herd management

Please, choose correct answer, only one answer is possible, unless stated otherwise.

1. **Region**____
2. **County**____
3. **No. of animals in herd:**____________
4. **Herd type:**

   [1] Tethered [2] Untethered [3] Other______________________________
5. **Farming type:**

   [1] Industrial [2] Biological [3] Other______________________________
6. **Can animal leave the farm building? area, pasture, e.t.c.)**

   [1] Yes (How?)______________________________________

   [2] No

Calves

7. **Place of calving**

   [1] Separate calving space

   [2] Sleep area

   [3] Other ______________________________________
8. **Age of calf at the separation from dam**

   [1] Right after birth

   [2] Other ______________________________________
9. **When is colostrum given after birth?**

   [1] Up to 2 h [2] 2- 3 h [3] 3- 4h [4] 4-12h [5] Over 12 h
10. **How much colostrum is given (in liters) on the first time?**

    [1] till 1 [2] 1-2 [3] 2-3 [4] 3-4 [5] Other______________________________
11. **How long is milk given to calves?**

    [1] up to one week [2] 1 - 2 weeks [3] 2-3 weeks [4] 3-4 weeks [5] 1 month [6] 2 months

    [7] 2-3 months [8] over 3 months [8] milk replacer (how long?)___________
12. **Are calves held in groups?**

    [1] Yes [2] No *(continue with 15. question)*
13. **At what age are calves moved to group?**

    [1] up to 1 week of age [2] 1-2 weeks old [3] 2-3 weeks old

    [4] 3-4 weeks old [5] 1 month old [6] 2 months old

    [7] Other ______________________________________
14. **How many calves are held in one group?**

    [1] 1-5 [2] 5-10 [3] 10-15 [4] 15+ [5] Other______________________________
15. **Do calves have diarrhea?**

    [1] Yes [2] No (*continue with 22. question*)
16. **At what age is diarrhea seen in calves? (***multiple choice question***)**

    [1] up to 7 day old [2] 7-14 day old [3] 14-30 day old [4] above 30 day old

    [5] Other______________________________________
17. **Is diarrhea treated?**

    [1] Yes [2] No
18. **Did diarrhea treatment help?**

    [1] Yes [2] No
19. **In which calendar month, does diarrhea flare up?**________________________
20. **How many precent of calves have diarrhea?**

    [1] 1-2% [1] 2-5% [2] 5-10 % [3] above 10%
21. **Are calves with diarrhea isolated?**

    [1] Yes (How long?)______________________________________

    [2] No

Walking area and pasture

22. **Can animals leave farm house for a walking area?**

    [1] Yes [2] No (*continue with 24. question)*

ID NR___________ Date________

23. Is there water body in the walking area, which animals have access to?

[1] Yes [2] No

24. Are animals pastured?

[1] Yes [2] No (*continue with 35. question)*

25. When does pasture season starts?

[1] March [2] April [3] May [4] Other____

26. When does pasture season ends?

[1] September [2] October [3] November [4] Other___________________________________

27. Which factors affect the start and end of pasture season?_________________________

____

28. How often is pasture paddock changed?

[1] Once a month [2] Every two months [3] Every three months [4] Not changed [5] Other_____________

29. Is there a waterbody in pasture? (*river, pond, lake, ditch*)

[1] Yes (which?) ____________________ [2] No (*continue with 32. question)*

30. How far away from pasture is this waterbody?____

31. Can animals access this waterbody?

[1] Yes [2] No

32. Is this waterbody connected to another waterbody? (*Flows to a river, lake, e.t.c.*)

[1] Yes (To where?) ____________________ [2] No

33. How do animals drink water in the pasture? (*multiple choice question*)

[1] Open waterbody (which?)____

[2] Drinking water

[4] None

[4] Other ____

Farm management

34. How is manure removed from the herd?

[1] Automatically [2] Manually (With what?)____

[3] Both

35. If manual manure cleaning is used, is the equipment cleaned?__________

____

36. How often is sleeping areas cleaned from manure?____

37. How often is individual calf pens/calf group pens cleaned of manure?________________

38. Please describe the location of the manure storage

[1] A pile next to herd [2] Open manure pit [3] Closed manure pit (lagoon)

[4] Other____

39. How manure/slurry is disposed of?

[1] Collected by a company

[2] Self use (Where is it used?) ____

[3] Sold ____

40. Is manure/slurry processed before its utilization/use?

[1] Yes (How?)____

____

[2] No

41. How often is manure/slurry removed from the farm area ______________________________

42. Are manure storages cleaned after manure removal/use (washed)?

[1] Yes (how?)_________________________________ [2] No

43. Are the calf boxes disinfected after their stay in the individual boxes and group boxes?

[1] Yes [2] No (*continue with 45. question*)

44. How are the individual boxes disinfected? ____

____

____

45. How are calf group boxes disinfected? ____

____

____

ID NR__________ Date________

46. Are the cattle dewormed?

[1] Yes [2] No

47. How often are cattle dewormed?

[1] 1x a year [2] 2x a year [3] Other ____

48. Has the presence of rodents been observed in the farm?

[1] Yes [3] No (*continue with 51. question*)

49. Is rodent control carried out in the farm?

[1] Yes [2] No

50. Type of rodent control (*multiple choice question***)**

[1] Poison [2] Mechanical [2] Cat [3] Other_____________________________

51. Is the staff provided with protective equipment (change of shoes)?

[1] Yes [2] No

52. Is the veterinarian and other "third" persons provided with a change of shoes or a disinfection mat when entering the herd?

[1] Yes [2] No

53. Have the employees had complaints about diarrhea of unknown origin that lasts longer than 3 days?

[1] Yes [2] No

54. Do employees have the opportunity to disinfect their hands?

[1] Yes [2] No

55. Is there a dry toilet in the herd area?

[1] Yes [2] No

Feed

56. What type of feed is used to feed animals? (*multiple choice question***)**

[1] Totally mixed feed [2] Hay [3] Fresh grass [4] Silage [5] Haylage

[6] Other_______________________________________

Farm surroundings

57. What is located within a radius of 500-1000m around the farm? ***(multiple choice question)***

[1] Houses [2] Road/Railroad [3] Meadow [4] Forest [5] Bushes

[6] Water reservoir (pond, lakes, river, e.t.c.) [7] agricultural land

58. Please estimate the distance to the nearest farm with more than 10 cows ____________

59. Is there a water reservoir (pond, lake, river, ditches) around the farm area?

[1] Yes (What type and how far?)____

[2] No

60. Are there other farm animals on the farm? (*multiple choice question)*

[1] Horses [2] Sheep [3] Goats [4] Pigs [5] Domestic birds [6] Rabbits [7] No

61. Are there other pets on the farm? (*multiple choice question*)

[1] Cats [2] Dogs [3] No [4] Other____

62. Have wild animals been observed in the vicinity of the herd/pasture?

[1] Yes (please fill in the table by ticking the appropriate time) [2] No

Wild animal species	**Time**				
	Every day	Once a week	Once every two weeks	Once a month	Less than once a month
Wild boar					
Wild ruminants					
Wild carnivores					
Wild birds					
